# Nuclear functions of β2-Spectrin in genomic stability

**DOI:** 10.18632/aging.101147

**Published:** 2016-12-14

**Authors:** Kalpana Mujoo, Clayton R. Hunt, Tej K Pandita

**Affiliations:** Department of Radiation Oncology, Weill Cornell Medical College, The Methodist Hospital Research Institute, Houston, TX 77030, USA

**Keywords:** β2-Spectrin, DNA damage response

Genomic instability is a hallmark of most cancers. Most critical factors regulating genomic stability reside in the nucleus and for a specific type of DNA damage, the double strand break, this is largely orchestrated by ATM (ataxia-telangiectasia mutated) [1], a serine /threonine kinase, that can be activated throughout the cell cycle by ionizing radiation (IR) exposure [2]. Individuals with ataxia-telangiectasia (A-T) are deficient in ATM and are characterized by, amongst other conditions, premature aging and genomic instability that results in a predisposition to cancer. Kirshner and coworkers reported that cells deficient in transforming growth factor-beta (TGFβ), a cytoplasmic factor, have impaired ATM activation that abrogates the DNA damage response [3]. New effectors of TGFβ are being identified and their relationship to DNA damage repair is evident.

The most common non-erythrocytic member of the β-spectrin gene family is the ubiquitously expressed β2-Spectrin (β2SP, gene Sptbn1). The principal adaptors of β2-Spectrin are the ankyrin proteins, which bind to the cytoplasmic domains of numerous integral membrane proteins [4], and the long-range connectivity of spectrin-actin network with Tropomodulin1 (Tmod1) and γ–tropomyosin (γ-TM) [5]. β2-Spectrin is a dynamic intracellular non-pleckstrin homology (PH)-domain protein that belongs to a family of polypeptides implicated in cell polarity. β2-Spectrin is an effector of TGFβ, which is a pleiotropic cytokine that regulates multiple cellular functions including proliferation, angiogenesis, and immune responses [6]. Although, β2-Spectrin is cytoplasmic factor, its new nuclear function in the DNA damage response indicates a role in events normally associated with nuclear defects, aging and cancer.

Horikoshi and co-workers revealed a role for the TGF-β effector β2-spectrin (β2SP) in maintaining genomic stability [7]. Both human and mouse cells deficient in β2SP show enhanced spontaneous genomic instability as well as moderate sensitivity to IR as determined by clonogenic survival assay. In contrast to IR response, extreme sensitivity to agents that cause interstrand cross-links (ICL) or replication stress was observed in cells deficient in β2SP. The enhanced cell killing in β2SP deficient cells could be due to defects in DNA damage sensing or DNA damage repair. In response to treatment with IR or ICL inducing agents, β2SP-deficient cells displayed a higher frequency of cells with delayed γ-H2AX foci disappearance and a higher frequency of chromosome aberrations, suggesting that depletion of β2SP resulted in defective DSB repair [7]. The reduced number of HR related repairosome foci post damage further supports the idea that β2SP may have a role in HR. Thus the genomic instability observed in β2SP-deficient cells is attributed to defective HR repair as a higher levels of S-phase specific IR-induced chromosome aberrations were also observed. Since HR based repair is upregulated in S-phase, increased S-phase specific aberrations are indicative that β2SP-deficiency impacts HR mediated DNA DSB repair. β2SP-deficiency also resulted in the higher frequency of 53BP1/RIF1 colocalization, support-ing the argument that HR is suppressed in β2SP-depleted cells. Following hydroxyurea-induced replication stress, β2SP-deficient cells displayed defective repair factor recruitment (Mre11, CtIP, Rad51, RPA, and FANCD2) as well as defective replication restart. These results support the argument that β2SP is required for maintaining genomic stability following replication fork stalling, whether induced by either ICL damage or replicative stress, by facilitating fork regression as well as DNA damage repair by HR. In addition β2SP depleted cells also showed reduced recruitment of RAD51 and MRE11 at l-Scel induced DSB [7], supporting the role of β2SP in DSB repair by HR.

Targeting β2SP may enhance IR-induced cell killing and thus improve radiotherapy. Alternatively, restoration of β2SP expression in normal tissue may suppress, by altering the Fanconi anemia DNA repair pathway, oncogenesis due to dysfunctional TGF-β/β2SP signaling impacted by the processing of genotoxic metabolites.
